# Genome Sequence of the Asian Honeybee in Pakistan Sheds Light on Its Phylogenetic Relationship with Other Honeybees

**DOI:** 10.3390/insects12070652

**Published:** 2021-07-16

**Authors:** Hongwei Tan, Muhammad Naeem, Hussain Ali, Muhammad Shakeel, Haiou Kuang, Ze Zhang, Cheng Sun

**Affiliations:** 1 Laboratory of Evolutionary and Functional Genomics, School of Life Sciences, Chongqing University, Chongqing 401331, China; thwhoneybee@163.com; 2 Chongqing General Station of Animal Husbandry Technology Extension, Chongqing 401121, China; 3 Department of Zoology, Faculty of Engineering and Applied Sciences, Faisalabad Campus, Riphah International University, Faisalabad 38000, Pakistan; dr.naeem@riphahfsd.edu.pk; 4 Entomology Section, Agriculture Research Institute D. I. Khan, Dera Ismail Khan 29120, Pakistan; hussaintanha@yahoo.com; 5 Department of Entomology, The University of Agriculture Peshawar, Peshawar 25120, Pakistan; shakeelkhanmarwat@yahoo.com; 6 Faculty of Animal Science and Technology, Yunnan Agricultural University, Kunming 650201, China; kuang68307@163.com; 7 Institute of Apicultural Research, Chinese Academy of Agricultural Sciences, Beijing 100093, China

**Keywords:** *Apis cerana*, genome sequencing, phylogeny

## Abstract

**Simple Summary:**

The Asian honeybee, *Apis cerana*, is used for honey production and pollination services in Pakistan. However, its genome sequence is still unknown. We collected *A. cerana* samples from its main rearing region in Pakistan and performed whole genome sequencing. We obtained a remarkably complete genome sequence for *A. cerana* in Pakistan, from which we identified a total of 11,864 protein-coding genes. Phylogeny analysis indicated an unexpectedly close relationship between *A. cerana* in Pakistan and those in China, suggesting a potential human introduction of the species between the two countries. Our results will facilitate the genetic improvement and conservation of *A. cerana* in Pakistan.

**Abstract:**

In Pakistan, *Apis cerana,* the Asian honeybee, has been used for honey production and pollination services. However, its genomic makeup and phylogenetic relationship with those in other countries are still unknown. We collected *A. cerana* samples from the main cerana-keeping region in Pakistan and performed whole genome sequencing. A total of 28 Gb of Illumina shotgun reads were generated, which were used to assemble the genome. The obtained genome assembly had a total length of 214 Mb, with a GC content of 32.77%. The assembly had a scaffold N50 of 2.85 Mb and a BUSCO completeness score of 99%, suggesting a remarkably complete genome sequence for *A. cerana* in Pakistan. A MAKER pipeline was employed to annotate the genome sequence, and a total of 11,864 protein-coding genes were identified. Of them, 6750 genes were assigned at least one GO term, and 8813 genes were annotated with at least one protein domain. Genome-scale phylogeny analysis indicated an unexpectedly close relationship between *A. cerana* in Pakistan and those in China, suggesting a potential human introduction of the species between the two countries. Our results will facilitate the genetic improvement and conservation of *A. cerana* in Pakistan.

## 1. Introduction

Among all honeybee species, the three species, *Apis cerana*, *A. florea*, and *A. dorsata*, are endemic to Pakistan. The traditional beekeepers have been using *A. cerana* for honey production for the last two centuries because of its ability to cope with environmental challenges [1,2]. Currently, the average honey production of *A. cerana* has ranged from 7 to 9 kg/colony, and on an annual basis, 60 tons of honey are produced from *A. cerana* in Pakistan [2,3]. *A. cerana* could also be used for pollination services to improve crop production [4]. The first time, PARC (Pakistan Agricultural Research Council) made an effort to use *A. cerana* as pollinators, which has also promoted increasing awareness among the farmers to use honeybee colonies as pollinators in Pakistan [5].

Currently, introduced honeybee species *Apis mellifera* are used for honey production and pollination services in Pakistan. However, such an introduction presents a severe threat to local biodiversity [6]. For example, in the 1980s, the tracheal mite attacking *A. cerana* might be due to the mite transfer from *A. mellifera* [2]. Hence, priority should be given to local species for honey production and pollination services, to avoid such incidents. *A. cerana* beekeeping is again being encouraged, especially in the mountainous regions where it is better adapted than *A. mellifera* and has no problems with *Varroa destructor* [1]. However, the genomic makeup of the commonly used *A. cerana* in Pakistan is still unknown, which will impede its improvement and conservation. In addition, the natural distribution of *A. cerana* occurs across southern and southeastern Asia, and extends to the Afghanistan region in the west [7]. There are different climatic conditions across the distribution range of this species, which will promote the genetic differentiation of *A. cerana*. However, little is known about the phylogenetic relationship between *A. cerana* in Pakistan and those in other countries.

In this study, we collected *A. cerana* samples from the main cerana-keeping region in Pakistan. We then generated and characterized its genome sequence, and based on this we inferred the phylogenetic relationship between *A. cerana* in Pakistan and those in other countries.

## 2. Materials and Methods

Drones of *A. cerana* were collected from one colony reared at an apiary located in Khyber Pakhtunkhwa, a main cerana-keeping region in Pakistan (34.0851029° E & 71.6149948° N), in 2018. Genomic DNA was purified from each drone with the use of a Qiagen Gentra Puregene Tissue Kit. To avoid contamination, the abdomen of each drone was removed before DNA extraction.

We took a genome sequencing and assembly method as described before [8]. In brief, one single drone of *A. cerana* was used to extract genomic DNA, based on which one fragment library (insert size: 450 bp) was generated by using NEBNext^®^ Ultra™ DNA Library Prep Kit for Illumina^®^ (NEB, Ipswich, MA, USA). The obtained fragment library was sequenced on the Illumina HiSeq 2500 platform (read length: 250 bp), from which overlapping paired-end shotgun reads (2 × 250 bp) were produced. Genomic DNA purified from five drones of the same *A. cerana* colony was used to produce large-insert jumping libraries with four different sizes (4 kb, 6 kb, 8 kb, and 10 kb, respectively), following a protocol as described before [9]. The obtained jumping libraries were sequenced on the Illumina HiSeq X Ten platform, with a read length of 150 bp, from which paired-end shotgun reads (2 × 150 bp) were generated. To produce genome assembly, at first, the 250 bp overlapping paired-end reads from the fragment library were assembled using the software DISCOVAR de novo v52488, an assembler performing well at assembling insect genomes [10], to produce continuous sequences (contigs). Then, shotgun reads from the four jumping libraries were used for scaffolding the contigs using the software BESST v2.2.6 [11]. Software BUSCO v3 [12] was employed to evaluate the completeness of the genome assembly by using 4415 Benchmarking Universal Single-Copy Orthologs (BUSCOs) (dataset: hymenoptera_odb9).

Protein-coding genes of the *A. cerana* genome were annotated by the MAKER computational pipeline, which was based on ab initio gene predictions, transcript evidence, and homologous protein evidence [13]. Ab initio gene prediction software includes SNAP [14], GENEMARK [15] and AUGUSTUS [16]. Transcript evidence came from predicted transcripts of *A. cerana* (GenBank: GCF_001442555.1_ACSNU-2.0_rna.fna) and *A. mellifera* (GenBank: GCF_003254395.2_Amel_HAv3.1_rna.fna). Protein evidence came from annotated Apoidea proteins that are publicly available in GenBank (last accessed on 29 January 2018).

To provide functional clues for the predicted protein-coding genes, the protein sequences that they encode were used as queries to do BLASTp (in BLAST suite v2.28 [17]) against the Swiss-Prot database (last accessed on 28 January 2018). In addition, program InterproScan-5 [18] was used to identify protein domains and GO terms that are associated with the predicted protein-coding genes. The quality of gene annotation was evaluated by comparing predicted protein sequences of the *A. cerana* genome to 4415 BUSCOs, using the software BUSCO v3 (lineage dataset: hymenoptera_odb9) [12].

Transposable elements (TEs) were de novo identified by RepeatModeller2 [19]. Using the repeat library obtained from RepeatModeller2, *A. cerana* genome assembly was analyzed with RepeatMasker (http://www.repeatmasker.org (accessed on 28 January 2018) to generate a summary of its TE landscape. Tandem Repeat Finder [20] was employed to identify tandem repeats in the *A. cerana* genome.

The predicted protein sequences for *A. mellifera* (GenBank assembly: GCA_003254395.2), *A. cerana* from Korea (GenBank assembly: GCA_001442555.1), *A. cerana* from China (GenBank assembly: GCA_002290385.1), *A. florea* (GenBank assembly: GCA_000184785.2), *A. dorsata* (GenBank assembly: GCA_000469605.1), and *Bombus terrestris* (GenBank assembly: GCA_000214255.1) were downloaded from GenBank. Proteins for *A. cerana* in Pakistan are from this study. Proteins for *A. laboriosa* were from the National Genomics Data Center, Beijing Institute of Genomics, Chinese Academy of Sciences (https://bigd.big.ac.cn/ (accessed on 21 October 2020); assembly accession: GWHAOTM00000000). Only the longest isoform of each protein-coding gene was kept for downstream analysis. Protein sequences of the eight sequenced bee genomes were used to infer orthologous groups (gene families) by using Orthofinder v2.4.0 [21].

To construct the molecular phylogeny for these seven honey bees that have a sequenced genome (*A. mellifera*, *A. cerana* from Korea, *A. cerana* from China, *A. florea*, *A. dorsata*, *A. laboriosa* and *A. cerana* from Pakistan), with one bumblebee (*B. terrestris*) serving as the outgroup, universal single-copy orthologs inferred by Orthofinder [21] were extracted. Protein sequences from those universal single-copy orthologs were multiply aligned with program MAFFT [22], respectively, followed by alignment trimming by software BMGE [23]. Alignments shorter than 50 amino acids or with more than 20% gaps were removed. The remaining alignments were concatenated for each of the eight bee species, resulting in eight super-sequences. Software ModelFinder [24] was used to select the best-fitting amino acid substitution model. IQ-TREE version 1.6.1 [25] and RAxML-NG version 1.0.2 [26] were employed to construct maximum likelihood trees for these eight bees, with 1000 bootstrap replicates, respectively.

## 3. Results

### 3.1. Genome Sequencing, Assembly and Annotation

Genomic DNA extracted from six males of the same *A. cerana* colony were used to construct one fragment library and four jumping libraries for whole genome sequencing (see Methods). We obtained a total of 22.67 Gb of overlapping paired-end shotgun reads (2 × 250 bp) from the fragment library (Table 1), which were used to produce contigs. A further 5.95 Gb of shotgun reads were obtained from the four jump libraries (Table 1), which were used to scaffold the contigs.

The obtained genome assembly had a total length of 214.44 Mb, with a GC content of 32.77%. The assembly had a scaffold N50 of 2.85 Mb and a contig N50 of over 311 Kb (Table 1). To evaluate the completeness of the resulting genome assembly, we compared genes present in the assembly to 4415 hymenopteran BUSCOs. We found that 99.5% of BUSCOs (99% of them are complete) were present in the genome assembly (Table 1), suggesting that we have obtained a remarkably complete genome sequence for reared *A. cerana* in Pakistan.

A total of 11,864 protein-coding genes were predicted using the MAKER pipeline (Table 1). Of them, 6750 genes were assigned at least one Gene Ontology (GO) term, and 8813 genes were annotated with at least one protein domain (Table 1). BUSCO analysis suggested a high-quality of genome annotation (Table 1). The assembled *A. cerana* genome contains 6.08% of repetitive sequences, and 0.7% of the genome are recognized as transposable elements (TEs). Mariner DNA transposons represent the most abundant TEs, consistent with previous reports for *A. cerana* in other countries [27,28,29].

### 3.2. Phylogeny Analysis of A. cerana in Pakistan

To understand the phylogenetic position of reared *A. cerana* in Pakistan, we constructed the genome-scale molecular phylogeny for seven honeybee genomes, including three Asian bees from different geographical regions (*A. mellifera*, *A. florea*, *A. dorsata*, *A. laboriosa*, *A. cerana* from Korea, *A. cerana* from China and *A. cerana* from Pakistan), with one bumblebee species (*B. terrestris*) serving as the outgroup. Protein sequences of these eight bees were downloaded and clustered, with single copy orthologs being delineated (see Methods). A total of 6101 single copy genes were identified across the eight bees and were multiply aligned. After trimming the alignments and getting rid of low-quality alignments, a total of 5234 single-copy genes remained, which were concatenated for each species, resulting in eight super-sequences. The super-alignment contained 2,980,871 amino-acid sites, with 44,493 distinct site patterns.

**Figure 1:**
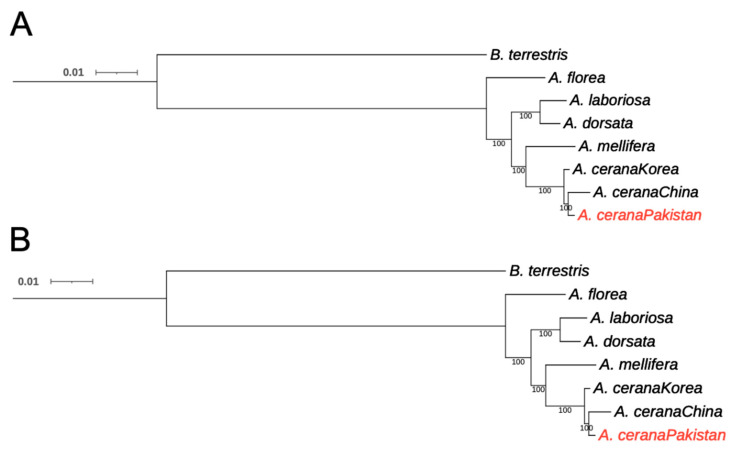
Maximum likelihood trees constructed by IQ-TREE (**A**) and RAxML-NG (**B**). Numbers on the nodes indicate bootstrap values.

IQ-TREE [25] and RAxML-NG [26] were used to construct maximum likelihood trees for the eight bees, with the best-fitting amino acid substitution model JTT+F+R3. Both softwares yielded exactly the same topology, with high supporting values (Figure 1). From the phylogeny, we could see that *A. cerana* in Pakistan consistently clustered with *A. cerana* from China with a short branch, which formed a sister group to *A. cerana* from Korea.

## 4. Discussion

At present, there are nine species that have been commonly recognized in the genus *Apis*, which including *A. andreniformis*, *A. cerana*, *A. dorsata*, *A. florea*, *A. koschevnikovi*, *A. laboriosa*, *A. mellifera*, *A. nigrocincta* and *A. nuluensis* [30]. Phylogenetic analyses using mitochondrial genes strongly supported the basic topology that groups the nine honeybee species into three major clusters: giant bees (*A. dorsata* and *A. laboriosa*), dwarf bees (*A. andreniformis* and *A. florea*) and cavity-nesting bees (*A. mellifera*, *A. cerana*, *A. koschevnikovi*, *A. nuluensis* and *A. nigrocincta*) [30,31] *A. mellifera* and *A. cerana* are both widely used for pollination services and honey production. Based on a recent analysis, *Apis* species originated from tropical regions, and out of the nine *Apis* species, only *A. mellifera* and *A. cerana* have expanded northward into the temperate zone, which may explain their large-scale application in agriculture [31].

In this study, we have generated a high-quality reference genome for *A. cerana* in Pakistan (Table 1), which will form a foundation for future research, including resequencing and population genomic studies for functional gene positioning and cloning, and thus will facilitate the genetic improvement and conservation of *A. cerana* in Pakistan. Besides the *A. cerana* genome in Pakistan that was generated in this study, there are two other *A. cerana* genomes with publicly available genome assemblies and annotations: one is for *A. cerana* samples collected in Korea [27], and the other is for *A. cerana* samples collected in China [28]. Phylogenetic analysis based on whole genomes will yield more accurate results than analysis based solely on mitochondrial genes, so these *A. cerana* genomes will be valuable for understanding the origin and evolution of bees.

Unexpectedly, we found a very close relationship between *A. cerana* in Pakistan and *A. cerana* in China (Figure 1). Pakistan represents the west end of the *A. cerana* natural distribution range [32], so it is not impossible that *A. cerana* in the two counties are similar. However, the Himalayan mountains lie between Pakistan and China, which could serve as a natural barrier to prevent the mixing of *A. cerana* between the two countries. In contrast, no such natural barriers exist between China and Korea. Therefore, it is quite un-expected that the phylogenetic relationship between the *A. cerana* of China and Pakistan is closer than that of China and Korea (Figure 1). Although phylogeny based solely on bee species with genome sequences could be incomplete, it might be also possible that *A. cerana* in Pakistan was introduced via trade or exchange from China, especially considering the fact that during 1980–1983, almost all of the *A. cerana* colonies kept in modern and traditional hives were killed by an epidemic of tracheal mites in Pakistan [2].

## Figures and Tables

**Table 1:** The results of genome sequencing, assembly and annotation for *A. cerana* in Pakistan.

Genome Sequencing				
	Read number (Million)	Read length (bp)	Total read length (Gb)	
Fragment library	90.66	250	22.67	
Jump libraries	39.65	150	5.95	
**Genome Assembly**				
Genome assembly size		Scaffold N50 (Mb)	Contig N50 (Kb)	BUSCO
214.44		2.85	311.13	99.50%
**Genome Annotation**				
Protein-coding gene number		Genes with a GO term	Genes with a protein domain	BUSCO
11,864		6750	8813	97.90%

## Data Availability

The raw Illumina shotgun reads and assembled genome sequence for *Apis cerana* in Pakistan have been deposited in National Center for Biotechnology Information (NCBI) under the BioProject number PRJNA669617.
